# Supplementary material for: Identification and functional analysis of non-coding regulatory small RNA FenSr3 in Bacillus amyloliquefaciens LPB-18
Source: PeerJ. 2023 May 15;11:e15236. doi: 10.7717/peerj.15236 (PMC10194069; doi:10.7717/peerj.15236)
Supplement: Supplemental Information 4 [file peerj-11-15236-s004.zip › KO/CK-vs-T1_map/map04750.html]

KEGG PATHWAY: Inflammatory mediator regulation of TRP channels - Reference pathway


|  |  |
| --- | --- |
| **Inflammatory mediator regulation of TRP channels - Reference pathway** |  |

[
Pathway menu
| Organism menu
| Pathway entry
| Show description
| User data mapping
]

The TRP channels that exhibit a unique response to temperature have been given the name thermo-TRPs. Among all thermo- TRP channels, TRPV1-4, TRPM8, and TRPA1 are expressed in subsets of nociceptive dorsal root ganglion (DRG) neuron cell bodies including their peripheral and central projections. These channels can be modulated indirectly by inflammatory mediators such as PGE2, bradykinin, ATP, NGF, and proinflammatory cytokines that are generated during tissue injury. While the noxious heat receptor TRPV1 is sensitized (that is, their excitability can be increased) by post-translational modifications upon activation of G-protein coupled receptors (GPCRs) or tyrosine kinase receptors, the receptors for inflammatory mediators, the same action appears to mainly desensitize TRPM8, the main somatic innocuous cold sensor. This aforementioned sensitization could allow the receptor to become active at body temperature, so it not only contributes toward thermal hypersensitivity but also is possibly a substrate for ongoing persistent pain.

|  |  |  |
| --- | --- | --- |
| Reference pathway | 184% 150% 122% 100% 82% 67% 55% | 图片下载 |
